# Socioeconomic Dynamics of Gender Disparity in Childhood Immunization in India, 1992–2006

**DOI:** 10.1371/journal.pone.0104598

**Published:** 2014-08-15

**Authors:** Ranjan Kumar Prusty, Abhishek Kumar

**Affiliations:** 1 International Institute for Population Sciences, Deonar, Mumbai, India; Institute for Health & the Environment, United States of America

## Abstract

**Background:**

Recent evidence indicated that gender disparity in child health is minimal and narrowed over time in India. However, considering the geographical and socio-cultural diversity in India, the gender gap may persist across disaggregated socioeconomic context which may be masked by average level. This study examines the dynamics of gender disparity in childhood immunization across regions, residence, wealth, caste and religion in India during 1992–2006.

**Method:**

We used multi-waves of the cross-sectional data of National Family Health Survey conducted in India between 1992–93 and 2005–06. Gender disparity ratio was used to measure the gender gap in childhood immunization across the selected socioeconomic characteristics. Multinomial regression analysis was used to examine the gender gap after accounting for other covariates.

**Result:**

Results indicate that, at aggregate level, gender disparity in full immunization is minimal and has stagnated during the study period. However, gender disparity – disfavouring female children – becomes apparent across the regions, poor households, and religion - particularly among Muslims. Adjusted gender disparity ratio indicates that, full immunization is lower among female than male children of the western region, poor household and among Muslims. Between 1992–93 and 2005–06, the disparity in full immunization had narrowed in the northern region whereas it had, astonishingly, increased in some of the western and southern states of the country.

**Conclusion:**

Our findings emphasize the need to integrate gender issues in the ongoing immunization programme in India, with particular attention to urban areas, developed states, and to the Muslim community.

## Introduction

Childhood immunization is one of the most cost-effective interventions to reduce the global burden of under-five deaths and thereby achieving the related Millennium Development Goals (MDG 4). Following the World Health Organization's initiative of Expanded Program of Immunization (EPI) 1974, several attempts were made to increase childhood immunization. As a result, at global level, childhood vaccinations have prevented more than 2 million deaths caused by the vaccine-preventable diseases (VPDs) [1]. However, VPDs such as measles, tetanus, pertussis, still account about 1.5 million (equal to 17 percent) of total under-five deaths, which is preventable by routine vaccination [2]. Moreover, about 22.4 million children were incompletely vaccinated at 12 months of age and remained at risk of vaccine-preventable morbidity and mortality. More than half of them are from India (32%), Nigeria (14%), and Indonesia (7%) [3].

India is one of the first countries which immediately adopted the Expanded Immunization Program in 1978 after its global initiation. In 1985–86, Government of India launched the Universal Immunization Programme (UIP) with much dynamism to attain at least 85 percent vaccination of all infants by 1990 against the six vaccine-preventable diseases identified by the UNICEF in 1984. The target to achieve the universal immunization was emphasised in subsequent health and population policies such as Child Survival and Safe Motherhood Programme, 1992; Reproductive and Child Health (RCH) Program, 1997; National Population Policy, 2000; and National Rural Health Mission, 2005–2017 [4], [5], [6].

Despite the continuous efforts, the country failed to achieve target of universal immunization coverage – only 54% of the children aged 12–23 months received the recommended doses of all the six vaccines [7]. Moreover, the coverage is largely skewed across the regions and socioeconomic status in the country. For instance, among the major states, full immunization coverage was as high as 82% in Tamil Nadu compared to as low as 30% in Uttar Pradesh; 36% among poorest people compared to 73% among richest people [7].

The stalled and uneven progress of childhood vaccination in India could be the result of a complex set of factors associated with the demand for and supply of the vaccines. Supply-related factors are clearly important; however, evidence suggests that an adequate supply of vaccines does not necessarily translate into children being vaccinated [8]. Factors affecting the demand for childhood vaccinations are more complex in nature. These include locality (urban/rural/slums) of residence [9], [10], [11], parental education [12], [13], socioeconomic status of the households [14], [15], [16], caste and religion [13], [17], [18], parity and mother's age at birth [19], [20], [21] and distance to health service centres [22], [23]. Gender discrimination – disfavouring female child – is also an important determinant of childhood vaccination in India [11], [20].

Indian society is overwhelmingly patriarchal. Discrimination against girl child starts from birth and spans during different life course. Persistent preference for a son is one of the strongest facilitators of gender discrimination in Indian society [24]. This has reflected in differences in the allocation of material goods, rights, opportunities and obligations between men and women [25], [26]. Favouring son over daughter are grounded in a number of economic, social and religious reasons, including financial support, old age security, property inheritance, dowry, family lineage, prestige and power, death rituals, and beliefs concerning religious duties [24], [27], [28], [29]. In contrast, a girl child is often perceived as a burden to her parents as they have to find a suitable groom for their girl and meet the marriage expenses with a large dowry.

The persistent and pervasive son preference in India results in discrimination against daughters that even extends to the provision for food and education [19], [28], [30], [31], [32]. Inclination for a son also manifests itself in sex-selective abortion and has resulted in a distorted child sex ratio (0–4 years) in the country [33], [34], [35]. Moreover, such gender-based discrimination disfavours the girl child from receiving proper nutrition, preventive care, treatment for illnesses and disease, the consequence of which is excess female mortality and poor health of girl children [11], [19], [27], [36], [37], [38], [39].

Girls born in India have a 40% higher risk of ill-health as compared to boys and are less likely to access healthcare services including immunization [40], [41]. Boys, however, are more likely than girls to die in the first month of life from perinatal conditions, such as birth asphyxia and birth trauma. Beyond these causes and contrary to the trends observed in most of the world; more girls than boys in India die due to acute respiratory, infectious & parasitic diseases, and viral infections [42], [43].

**Figure 1:**
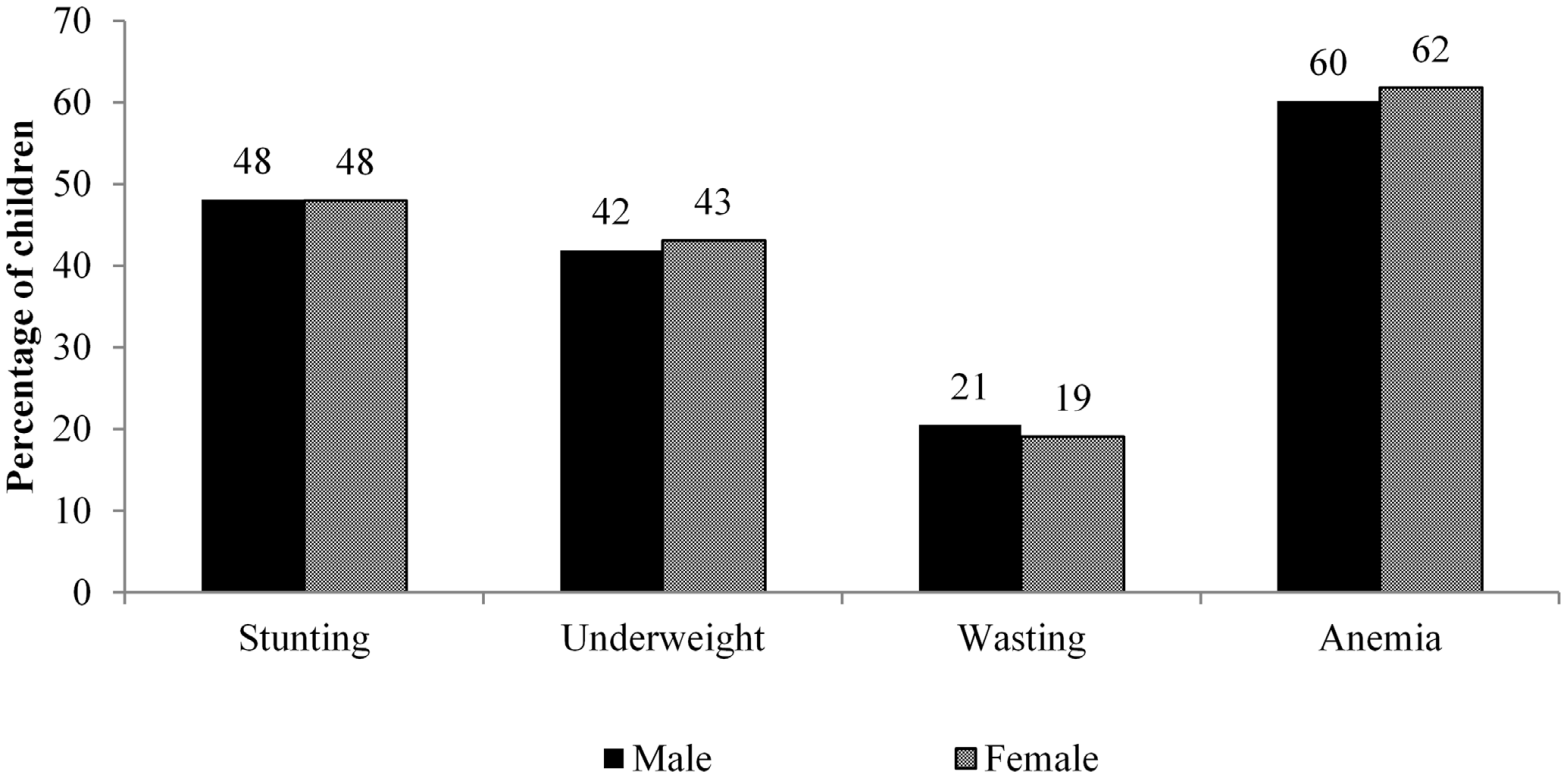
Gender gap in selected health indicators among children aged less than five years in India, 2005–06.

Gender gap in immunization coverage has been shown to exist in all states of India [11], [30]. These studies showed that female children are significantly less likely to receive full immunization compared to their male counterpart. A few other studies also indicated lower immunization coverage among girls as compared to boys, but differences were insignificant [44], [45]. The evidence from recent national-level data shows that the gender gap in full immunization coverage has either stagnated or narrowed [14], [46]. Moreover, estimates from the latest round of the Demographic Health Survey of India show that average gender gap in child health is minimal in the country (Figure 1). However, this average result may mask the disaggregated scenario owing the high diversity that exists across regions, socioeconomic, demographic and cultural contexts in the country [47].

In India, gender preferences vary across states, socioeconomic and cultural milieu [48]. One could reasonably expect a varying gender gap in immunization coverage across the socioeconomic and cultural contours. However, little is known about patterns in the gender gap that exist in immunization coverage across socioeconomic contexts and time. In this study, we examined the gender gap in childhood immunization across regions, place of residence, household wealth quintiles, caste, and religious groups in India between 1992–93 and 2005–06.

There is a clear North-South distinction in gender inequality, which is mainly determined by differing kinship patterns and marriage norms – influencing factors for differing gender norms – between these two broad regions [48], [49]. Additionally, the agro-economic occupational compulsions of the region also reflect the gender norms for that region. The higher demand for women labour in rice-growing areas makes girls and women more valuable than in the areas where wheat is cultivated. Hence, there is less discrimination against girls in rice-growing regions [48]. There is also less evidence of preference for male children in poor households. Here, women may be considered as economic assets for livelihood reasons; hence the poor households may be less able than wealthier households to enforce seclusion of women. Thus, they are allowed to play a more active economic role than they would be in wealthier households [39], [48].

Religion also plays a crucial role in defining social and gender norms which, in turn influence the preference for sons. The Hindus belief that son is necessary to carry the family's name forward and to perform the rituals after the death of his parents [50]. A recent study documenting son preference among various religious groups in India found that women from Muslim households have a slightly higher son preference. Women from other non-Hindu, non-Muslim religions, have slightly lower preference for sons than the Hindu and Muslim women [24]. Like religion, caste is also associated with cultural practices that influence women's roles and, thereby, preference for male children. One may expect that the lower castes and tribes may exhibit lower son preference than among other castes [24]. Compared to the traditionally ‘lower’ castes, higher castes have more rigid gender stratification systems with strictly enforced rules for seclusion for women [51]. Lower caste and tribal women may have fewer restrictions on their movement or employment outside the home [48], [52], often due to economic pressures that compel them to work for income.

In the light of such varying gender norms, the present study examines the gender disparity in immunization coverage in various socioeconomic contexts in India using data from the cross- sectional and multi-rounds of the National Family Health Survey conducted between 1992 and 2006. Context-specific evidence of gender disparity in immunization coverage may not only be helpful to the programmes that aim at achieving universal coverage of immunization; it may also help to minimize the gender gap in childhood mortality as well as improving overall child health. To the best of our knowledge, none of the previous studies has examined the pattern in gender gap in immunization coverage in the selected contexts.

## Methods

### Ethical Statement

The three waves of National Family Health Survey (NFHS) were conducted under the supervision of the International Institute for Population Sciences (IIPS), Mumbai, India, which serves as a regional institute for training and research in population studies for the ESCAP region. The ORC Macro institutional review board approved the data collection procedures. Formal written consents were obtained from the respondents and ethical issues were resolved before the respondents were interviewed. This study is based on anonymous public use datasets with no identifiable information about the survey participants. Survey data are available upon request on the official website of the institute at <www.measuredhs.com/data/dataset/India_Standard-DHS_2006.cfm?flag=0>.

### Data

Data for this study was taken from three successive rounds of the National Family Health Survey (NFHS) conducted between 1992–93 and 2005–06. The first round of NFHS was conducted in 1992–93, the second round in 1998–99 and the third in 2005–06. For convenience, we refer to the period between 1992–93 and 1998–99 as 1992–1998, the period between 1998–99 and 2005–06 as 1998–2005; while between 1992–93 and 2005–06 as 1992–2006. The NFHS is a household-based survey spanning the states of India. The main purpose of the survey is to provide reliable information on fertility, family planning, childhood mortality, maternal healthcare services, and childhood nutrition and immunization coverage at national and sub-national level.

All three rounds of the NFHS adopted multi-stage sampling design – two-stage sampling design in rural areas and three-stage in urban areas. The rural sample was selected in two stages: at first stage, Primary Sampling Units (PSUs), i.e., villages, were selected using probability proportional to size (PPS) sampling; and at second stage, required households were systematically selected within each PSU. In urban areas, at first stage, wards were selected using the PPS sampling. At second stage, a Census Enumeration Block (CEB) was selected by PPS from each selected ward; and at third stage, households were randomly selected within each selected CEB. The sampling design remained similar in all three rounds of the survey which allows a comparison of the estimates of the consecutive rounds [32], [53]. All three rounds of the NFHS collected data using different interview schedules i.e. household schedule and eligible women/individual schedule. The contents of interview schedule remained similar in all the three rounds. The NFHS-1 covered a sample of 89,777 ever-married women aged 13–49, NFHS-2 covered 90,303 ever-married women aged 15–49 and NFHS-3 covered 124,385 women (unmarried and married) aged 15–49. The individual response rate was 96 percent in the first and second rounds, while it was 94 percent in the third round of the survey [54], [55], [56].

### Outcome variable

Childhood immunization is used as the outcome variable in the study. The information on immunization was collected from mother (women) with at least one child aged less than five years. Immunization status is estimated using the information based on health card of the index child. In the absence of the immunization card, the information gathered from mother of the respective child. This is the standard practice for measuring immunization status using the large-scale population-based survey [57], [58]. There were variation in number of live births and reference period in which information on vaccination was collected. In NFHS-1, information on vaccination was collected for the last three births in the four years preceding the survey date; in NFHS-2, it was last two births in the three years preceding the survey date; whereas in NFHS-3, information was collected for last five births in the five years preceding to the survey date. Therefore, to make the estimate comparable over time, we considered last two births in the three years preceding the surveys date. Furthermore, immunization coverage is estimated for children aged between 12–23 months only, following the WHO guidelines. During the NFHS–1, the final analytical sample size was 11,602 children (last two births in three years preceding the survey and aged 12–23 months), in NFHS–2, the sample size was 10,209 children; and in NFHS–3, the sample was 9,582 children. The term ‘immunization’ and ‘vaccination’ is used interchangeably in this paper.

Immunization of a child is categorised into three categories: *full immunization*—surviving children who have received one dose of BCG, three doses of DPT vaccine, three doses of polio vaccine, and one dose of measles vaccine; *partial/any immunization*—surviving children who have received at least one vaccine; and *no immunization*—surviving children who did not receive any vaccine. This study focused on *full* and *no immunization* only since these are the two most important indicators of the effectiveness of the functioning of the health system perspective [11].

### Main predictors

The main predictors used in the study were: region, place of residence, household wealth quintile, caste, and religion. We selected these variables because gender norms as well as immunization coverage varied across these entities.

#### Region

We followed the regional classification of NFHS [56]. The six regions are: North (Jammu and Kashmir, Himachal Pradesh, Punjab, Uttaranchal, Haryana, Delhi, Rajasthan), East (Bihar, Jharkhand, Orissa, West Bengal), Central (Chhattisgarh, Madhya Pradesh, Uttar Pradesh), Northeast (Assam, Arunachal Pradesh, Manipur, Meghalaya, Mizoram, Nagaland, Tripura), West (Gujarat, Maharashtra, Goa), and South (Andhra Pradesh, Karnataka, Kerala, Tamil Nadu). Sikkim and Nagaland were excluded from the analysis because NFHS-1 and NFHS-2 did not collect immunization data in these two states.

It is important to mention here that the NFHS-3 samples also included three newly-created states: Jharkhand, Uttaranchal, and Chhattisgarh. These states were respectively carved out of Bihar, Uttar Pradesh, and Madhya Pradesh in the year 2000. In the third round of the survey, data for the new states were generated by separating districts that were transferred from the erstwhile states to the new ones [56]. Using the same district codes, we created data for the states of Jharkhand, Uttaranchal, and Chhattisgarh for first and second rounds of the survey. It was necessary to make the regional estimates comparable over the study period. Previous studies in India showed that coverage of childhood immunization varied starkly across states [11], [14].

#### Place of residence

The NFHS followed the Census of India definition to demarcate the urban and rural areas in the survey for this study [59]. Evidence from India and outside documented that immunization coverage is significantly lower in rural areas [11], [60].

#### Household wealth quintile

The first and second rounds of the NFHS computed Standard of Living Index (SLI) based on an arbitrary scoring of household economic indicators such as housing quality, household amenities, size of landholding, and consumer durables etc. The SLI was divided into three categories – low, medium, and high. In the third round of the NFHS, a wealth index was computed by using Principal Component Analysis (PCA) and this index was divided into five quintiles (poorest, poorer, middle, richer, and richest). This wealth index is increasingly used to measure household economic status in large-scale household surveys [61], [62], [63].

In the present study, therefore, a separate wealth index, which is based on a set of selected household economic proxies, is computed for the first and second rounds of the NFHS by using the Principal Component Analysis. The wealth index was subsequently divided into five quintiles – poorest, poorer, middle, richer, and richest. This was done to make the estimates comparable over all three NFHS rounds. Several studies have shown that children belonging to the poorest households were significantly less immunized than children of wealthiest households [11], [14], [64].

#### Caste

Caste of a respondent was based on the women's self–identification as belonging to Scheduled Castes (SCs), Scheduled Tribes (STs), Other Backward Classes (OBCs), and other castes. The NFHS-1 did not provide information on OBCs, therefore we excluded this group from analysis. In the present analysis, we clubbed SCs and STs together and named as SCs/STs and the other caste as “others”.

#### Religion

Religious groups were divided into three categories: Hindus, Muslims, and Others (religious groups that were neither Hindu nor Muslim). A study from India reports that Muslim children are less likely to receive the childhood vaccination than Hindus and other religions [18].

### Covariates

The gender disparity in childhood immunization across the selected predictors was assessed after accounting for the following covariates: birth order of index child, mother's age at birth of the child, mother's exposure to media, current working status of mother, father's education, use of antenatal care, and possession of health card.

### Statistical analysis

Descriptive analysis is used to understand the level and pattern of childhood immunization in India across the selected predictors and also to examine the extent of gender disparity in coverage of full immunization and no immunization over the period.

### Measuring gender inequality in immunization

Gender differentials are calculated as a simple ratio of immunization rates for boys and girls, multiplied by 100, as follows:

Gender disparity ratio (GDR) for full immunization:
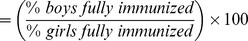



Gender disparity ratio (GDR) for no immunization:
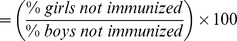



A GDR value of 100 indicates that there is no gender differential. The ratio for no immunization is inverted as compared to that for full immunization so that the interpretation of the ratio is consistent — GDR values exceeding 100 indicate that the girl child is at a disadvantage [11].

### Multivariate analysis

Multivariate analysis is used to examine the gender gap in childhood immunization across the selected predictors after accounting for the other covariates. As our dependent variable is nominal and consists of three outcomes — no immunization, partial immunization, and full immunization, we have used multinomial logit model on the pooled data of all the three rounds of the NFHS. The logit model allows the effects of the independent variables to differ for each outcome, and handles the non-independence of the categories of the dependent variable by simultaneous estimation of the models for all outcomes. The models were run separately for males and females. In order to make the interpretation more convenient, the result obtained from the multinomial regression analysis was converted into adjusted percentages using the Multiple Classification Application (MCA). The adjusted percentages were further used to compute the adjusted gender disparity ratio for full and no immunization across the selected predictors.

## Results

The overall coverage of full immunization among Indian children increased from 35% to 44% from 1992–93 to 2005–06 (Table 1). Coverage of partial/any immunization increased from 35% to 51%, while coverage of no immunization has decreased sharply from 30% to 5% during the period. The coverage of full, any, and no immunization across the selected characteristics are given in Table 1.

**Table 1:** Bivariate distribution of childhood immunization among children aged 12–23 months across the selected characteristics in India, 1992–2006.

	1992–93			1998–99			2005–06		
Characteristics	No Immunization	Any Immunization	Full Immunization	No Immunization	Any Immunization	Full Immunization	No Immunization	Any Immunization	Full Immunization
Region									
North	28.9	28.6	42.5	15.0	45.6	39.4	6.6	47.4	46.0
Central	41.2	36.9	21.9	25.4	55.0	19.6	3.8	67.5	28.7
East	39.6	38.2	22.1	14.6	58.9	26.5	7.0	48.4	44.6
Northeast	44.4	36.1	19.5	31.2	48.2	20.5	15.3	50.6	34.1
West	11.3	29.4	59.3	3.4	31.4	65.2	3.4	42.6	54.0
South	12.1	34.1	53.8	3.8	30.9	65.4	3.7	36.3	60.0
Place of residence									
Urban	16.4	32.9	50.7	6.3	38.9	54.8	3.5	38.9	57.6
Rural	34.0	35.1	30.9	16.8	48.4	34.9	6.0	55.5	38.6
Household wealth quintile									
Poorest	47.0	35.4	17.6	26.4	54.9	18.7	9.4	66.2	24.4
Poorer	38.6	37.2	24.2	20.0	53.6	26.4	6.3	60.5	33.2
Middle	32.8	36.1	31.0	15.4	45.7	39.0	4.8	48.4	46.9
Richer	21.9	35.9	42.2	7.3	42.6	50.0	2.9	41.8	55.3
Richest	10.8	28.5	60.7	3.6	34.9	61.5	1.0	28.1	71.0
Caste									
SCs/STs	39.3	34.9	25.8	18.1	48.2	33.7	7.6	55.4	37.0
Others	27.3	34.5	38.2	12.5	45.4	42.1	4.2	49.7	46.1
Religion									
Hindu	28.6	35.3	36.0	13.4	46.6	40.0	4.6	51.0	44.4
Muslim	41.1	32.6	26.3	21.1	49.1	29.8	8.0	55.7	36.3
Others	18.5	28.5	52.9	9.8	30.9	59.3	7.2	36.7	56.1
Overall	30.0	34.6	35.4	14.4	46.2	39.4	5.3	51.1	43.5

### Gender disparity in immunization coverage in India

**Figure 2:**
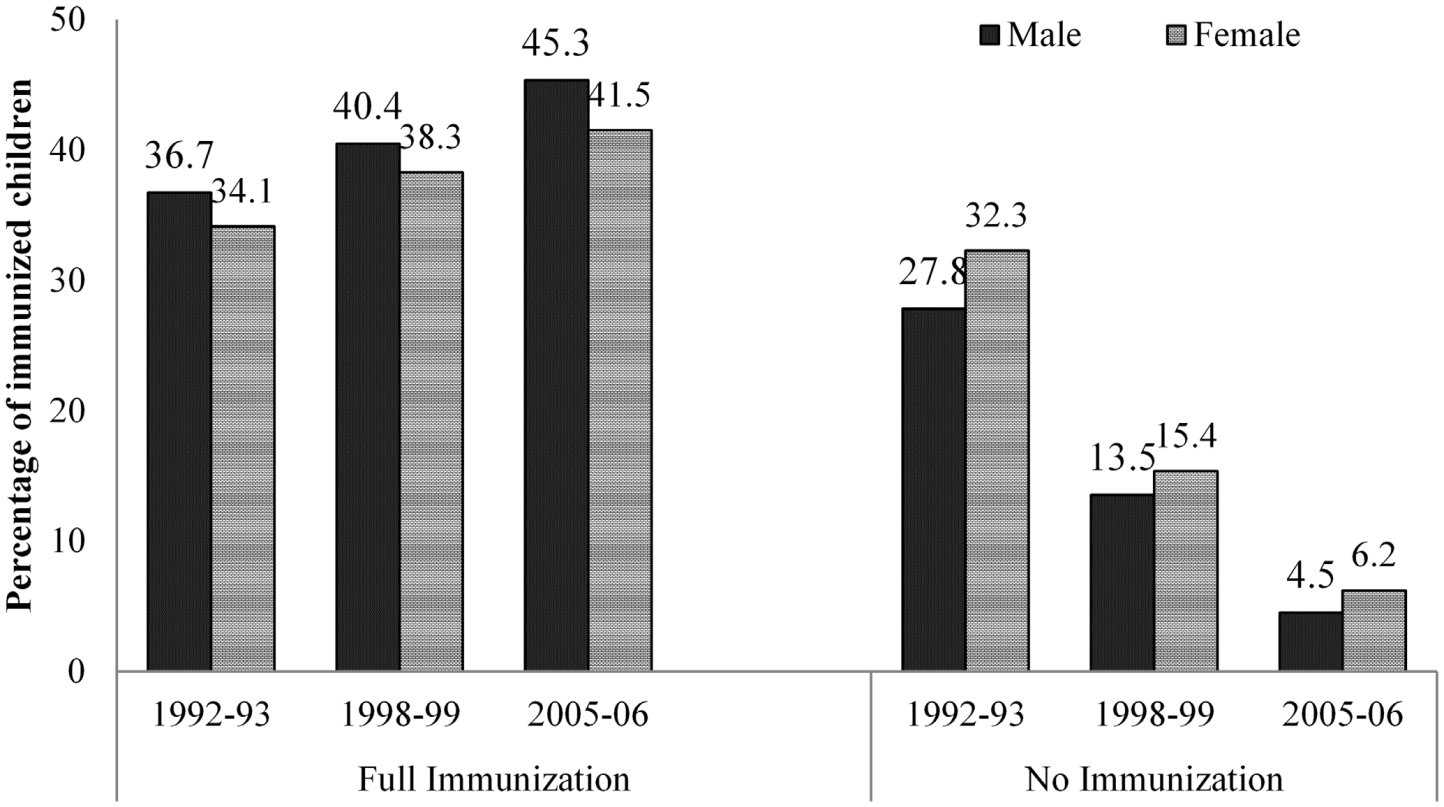
Differences in coverage of immunization status among male and female children in India, 1992–2006.

During 1992–2006, coverage of full immunization has improved among both male and female children, which was accompanied by a decline in the percentage of no immunization in this period (Figure 2). Among the male children, full immunization coverage increased from 37% to 45% and among female children, it has increased from 34% to 42% during the period. Coverage of no immunization declined sharply from 28% to 5% among male and 32% to 6% among female children during the period. Figure 2 also indicates that gender discrimination – disfavouring the girl child – is apparent in coverage of full and no immunization. Full immunization coverage was three percentage points higher among male than female children. The disparity in no immunization was minimal. The gender differences in the level of full and no immunization by different socioeconomic characteristics are not discussed in the text but for the convenience, readers may refer Appendix S1.

**Figure 3:**
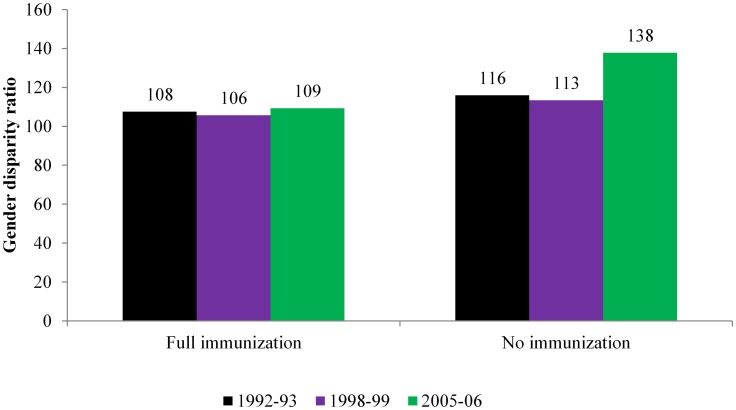
Gender disparity ratio in coverage of full and no immunization in India, 1992–2006.

The result of gender disparity ratio indicates that the female child is persistently disadvantaged in coverage of full immunization (Figure 3). The gender disparity ratio declined from 108 in 1992–93 to 106 in 1998–99. However, it again increased to 109 in 2005–06. GDR increased drastically in coverage of no immunization. It is important to mention here that this may be due to the extreme low level of no immunization among both male and female children.

#### Gender disparity by state/region

**Figure 4:**
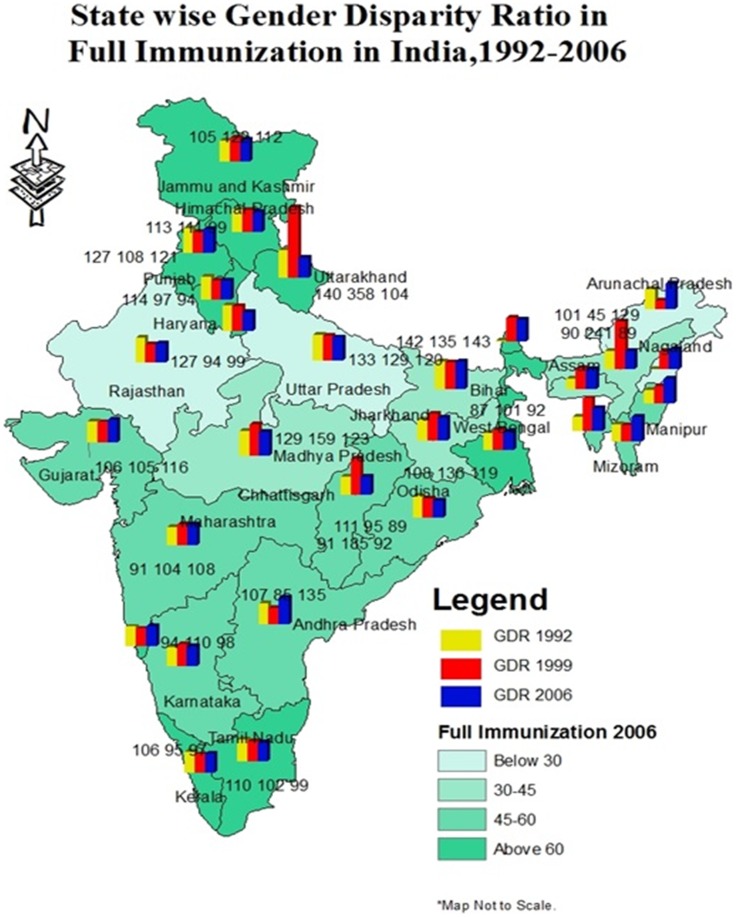
State wise gender disparity ratio in coverage of full immunization in India, 1992–2006.

**Figure 5:**
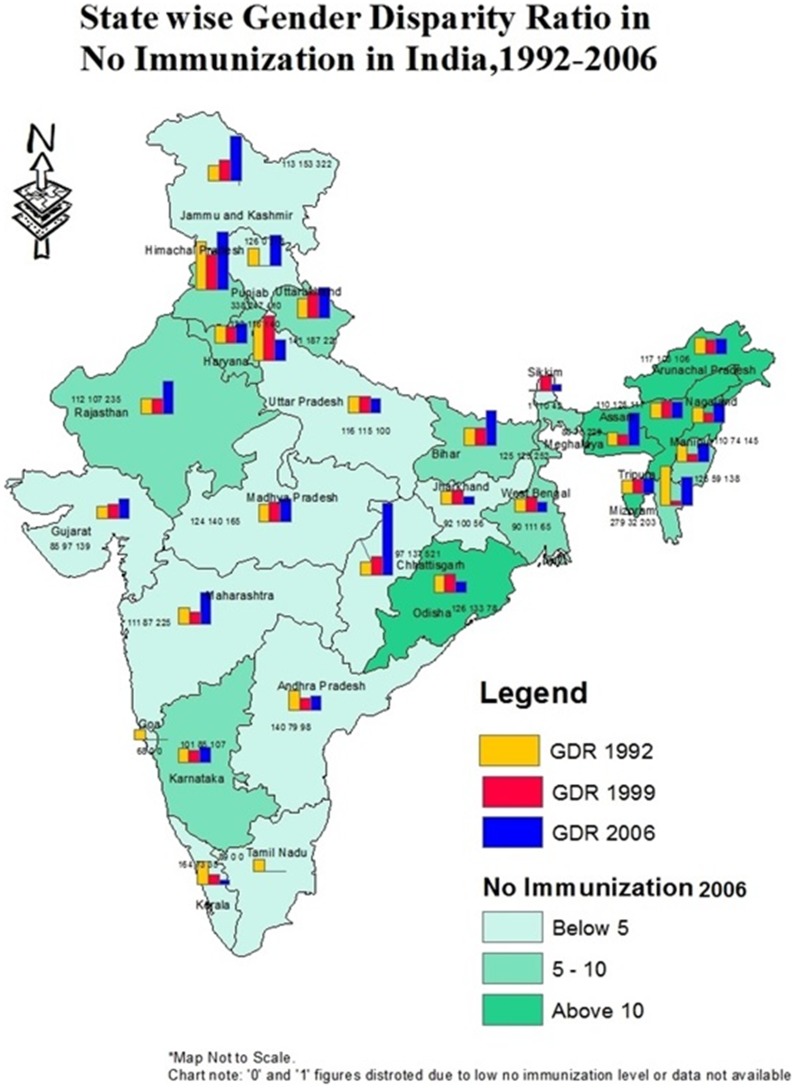
State wise gender disparity ratio in coverage of no immunization in India, 1992–2006.

At present, gender disparity ratio in full immunization is high in the state of Bihar (143), Andhra Pradesh (135), Arunachal Pradesh (129), Manipur (126), Mizoram (124), Madhya Pradesh (123), Punjab (121) and Uttar Pradesh (120) (Figure 4). However, going by trends, the results show that gender disparity has constantly increased in the state of Maharashtra during the period of 1992–2006. The disparity ratio has increased from 91 in 1992–93 to 104 in 1998–99 and to 108 in 2005–06 in Maharashtra. The details of the change in gender disparity ratio of full immunization by states have been shown in Figure 4. The GDR of no immunization have constantly gone up in the state of Bihar, Chhattisgarh, Jammu and Kashmir, Tripura and Uttaranchal (Figure 5).

While looking across the regions, result shows that female children were consistently in a disadvantageous position in full immunization coverage in most of the regions except the Northeast (Table 2). Gender disparity – measured by gender disparity ratio – in full immunization was comparatively higher in West, South, and Central regions of the country. The gender gap decreased in North and Central region while it has increased in the West and South region. During 1992–2006, the GDR declined from 124 to 107 in the North region, from 130 to 115 in the Central region. In contrast, the GDR increased from 95 to 112 in the West region and 104 to 110 in the South region during the same period. The disparity in no immunization also increased in the North, Central, East, Northeast and West regions during 1992–2006.

**Table 2:** Gender disparity ratios in full and no immunization among children aged 12–23 months across the selected characteristics in India, 1992–2006.

Backgrounds	Full Immunization			No Immunization		
	1992–93	1998–99	2005–06	1992–93	1998–99	2005–06
Region						
North	124	112	107	131	132	224
Central	130	133	115	117	114	123
East	103	106	105	116	122	120
Northeast	87	151	99	109	108	126
West	95	102	112	95	88	183
South	104	99	110	120	88	104
Place of residence						
Urban	97	100	106	86	105	146
Rural	112	109	110	120	115	136
Household wealth quintile						
Poorest	111	110	113	116	117	114
Poorer	115	101	97	121	104	177
Middle	115	104	110	116	109	118
Richer	103	110	114	103	134	155
Richest	102	104	98	116	128	103
Caste						
SCs/STs	105	112	112	116	114	142
Others	108	102	105	116	114	132
Religion						
Hindu	107	105	107	119	120	152
Muslim	107	109	115	105	91	95
Others	110	105	120	140	119	206

#### Gender disparity by place of residence

The gender gap in full immunization coverage is smaller in urban than in rural areas (Table 2). For example, GDR was 106 in urban areas and 110 in rural areas during 2005–06. Despite the lower disparity, the gender gap for full immunization increased in urban area from 97 to 106 during 1992–2006. During the same period, it decreased in rural areas from 112 to 110. The gender disparity also persists in coverage of no immunization. The GDR in no immunization was 146 in urban areas and 136 in rural areas in 2005–06.

#### Gender disparity by household wealth quintile

Gender disparity favoured the male child among the poorest, middle and richer wealth quintiles, while it was in favour of the female child among the poor and richest quintiles in 2005–06 (Table 2). GDR in full immunization increased among richer wealth quintile (103 to 114) while it has decreased among poor (115 to 97), middle (115 to 110) and richest (102 to 98) wealth quintiles during 1992–2006. The disparity in no immunization was highest among poor quintile (177) and lowest among richest quintile (103) during 2005–06. However, the disparity decreased in the two extreme quintiles (poorest and richest) while increased in the remaining quintiles.

#### Gender disparity by caste and religion

The GDR in full immunization was consistently higher among SCs/STs children as compared to other caste groups (Table 2). Moreover, the disparity has increased (105 to 112) among SCs/STs while decreased (108 to 105) among other caste groups during 1992–2006. The current level of gender disparity in no immunization was higher among SCs/STs (GDR 142) as compared to other caste (GDR 142). However, the disparity increased in both the caste groups during the study period.

Gender disparity in full immunization was higher among Muslims and other religious groups than among Hindus during 2005–06. It was also observed that the disparity stagnated among Hindus while it has increased among Muslims and other religion during 1992–2006. GDR remained constant (107) among Hindus during 1992–2006; among Muslims it has increased from 107 to 115, while among other religion it has increased from 110 to 120, during the same period. In case of no immunization, we observed a sharp variation in the gender gap across the religious groups: the GDR in no immunization is much higher among the other religion (206) and Hindus (152) than Muslims (95). Moreover, gender discrimination in no immunization has gone down among Muslims whereas it increased among Hindus and other religious group during the period.

### Multivariate analysis

The results obtained from bivariate analysis indicate gender disparity in coverage of full immunization across geographical, socioeconomic, and cultural contexts. However, this may lead to a biased conclusion as all the variables were not taken together. To that effect, we used multinomial regression analysis on the pooled data of all the three NFHS rounds, where all the selected predictors were adjusted along with other covariates. We ran the models separately for male and female children. In order to avoid complexity of interpretation, the results obtained from multinomial logit regression are presented in terms of predicted percentage using the MCA. Table 3 presents the predicted percentage of full immunization and Table 4 presents predicted percentage of no immunization among male and female by the selected socioeconomic characteristics in India.

**Table 3:** Results of multinomial logit regression (predicted percentages): full immunization coverage across male and female by selected characteristics in India, 1992–2006.

Covariates	1992–93		1998–99		2005–06	
	Male	Female	Male	Female	Male	Female
Region						
North*(Ref.)*	49.0	39.7	43.6	38.8	49.8	46.6
Central	24.3**	19.7**	22.3***	16.3***	30.9***	26.8***
East	24.1***	21.9***	27.4	26.5	44.4***	43.2
Northeast	19.5***	20.5***	26.6***	20.7***	38.9***	39.2***
West	57.2***	57.2***	61.0***	62.3***	57.4	52.5
South	53.4***	52.1***	66.2***	66.8***	62.6	57.3
Place of residence						
Urban*(Ref.)*	51.5	51.0	56.2	55.8	60.0	55.3
Rural	32.5	29.1	36.2**	33.8	40.4***	37.6*
Household wealth quintile						
Poorest*(Ref.)*	18.2	17.3	19.5	17.2	24.8	23.1
Poorer	25.3	22.6	25.6	25.6	32.8**	34.0
Middle	32.8	29.0	38.6	37.3	47.9*	45.5***
Richer	43.5	41.0	53.6***	50.1**	60.1***	54.1***
Richest	62.6	59.2	64.0**	62.0**	73.1***	71.7***
Caste						
SCs/STs*(Ref.)*	27.7	25.7	34.1	31.3	39.2	35.7
Other	38.5*	36.5***	43.4	42.2	48.4	45.2
Religion						
Hindu*(Ref.)*	37.1	34.5	41.1	39.3	46.9	43.8
Muslim	25.6***	24.9*	31.7***	30.3***	36.1***	31.4***
Others	50.4	51.1***	60.4	59.6	56.3	51.5

Ref.: Reference category;

***p<0.01;

**p<0.05;

*p<0.10.

The models are adjusted for birth order of index child, mother's age at birth of the child, mothers' exposure to media, current working status of mother, father's education, use of antenatal care, and possession of health card.

**Table 4:** Results of multinomial logit regression (predicted percentages): no immunization coverage across male and female by selected characteristics in India, 1992–2006.

Background	1992–93		1998–99		2005–06	
	Male	Female	Male	Female	Male	Female
Region						
North	23.9	32.5	12.8	17.6	6.1	11.6
Central	37.9	44.2	21.4	25.6	2.8	4.4
East	36.1	42.7	12.7	15.5	8.1	7.5
Northeast	43.0	48.1	30.6	32.7	13.6	14.3
West	12.6	12.7	3.5	3.2	2.3	4.6
South	11.6	13.5	4.0	3.5	3.4	3.5
Place of residence						
Urban	16.9	15.3	5.8	6.2	3.3	4.2
Rural	31.0	37.4	14.7	17.2	5.4	7.0
Household wealth quintile						
Poorest	43.9	50.9	22.8	27.8	9.7	10.6
Poorer	35.7	41.8	18.6	19.9	5.0	8.2
Middle	30.8	35.5	14.3	15.1	4.8	5.0
Richer	20.2	23.2	5.2	7.8	2.3	2.8
Richest	9.8	11.6	3.3	3.8	0.8	1.2
Caste						
SCs/STs	37.2	42.5	16.4	18.9	6.2	8.3
Other	26.4	29.4	11.2	12.8	4.3	5.4
Religion						
Hindu	26.7	31.3	11.3	14.0	4.1	5.6
Muslim	42.6	41.9	21.1	19.2	8.8	9.1
Others	21.5	23.2	9.0	9.8	5.0	9.2

The models are adjusted for birth order of index child, mother's age at birth of the child, mothers' exposure to media, current working status of mother, father's education, use of antenatal care, and possession of health card.

Full immunization coverage was significantly lower among females (27%, p<0.01) than male (31%, p<0.01) children in the Central region of the country during 2005–06 (Table 3). The gender gap was also observed in North (46% for female children as against 50% for male), West (53% and 57% for females and male children respectively), and South (57% for female children and 63% for males) region. However, the results were not significant consistently.

Gender gap in full immunization is also present across urban and rural residence; however, the gap was significant in rural areas only. During 2005–06, full immunization coverage was 55% among female and 60% among male children in urban areas. The corresponding figure in rural areas was 38% (p<0.10) and 40% (p<0.01). Gender gap was also pervasive across the household wealth quintiles. The disparity was significant among the middle (46% among female vs. 48% for male children), richer (54% among female vs. 60% among male), and richest wealth quintiles during 2005–06. A similar pattern was observed over the periods.

Gender differences in full immunization coverage were also observed within the caste groups. Among the SCs/STs, the coverage of full immunization was 36% among female children and 39% among male children during 2005–06. The corresponding percentages were 45% and 48% among children of other caste groups. The gender gap was also observed among Hindus (44% among female vs. 47% among male), Muslim (31% among female vs. 36% among male), and other religions (52% among female vs. 56% among male). However, the gap is significant only for Muslims over the time.

The gender gap in coverage of no immunization was observed in all six geographical regions of India (Table 4). It was more pronounced in the North region – in 2005–06, 12% of the female children were not immunized at all as compared to only 6% of male children. The gap was slightly higher in the rural than urban areas. It was also higher among the poor than other wealth quintiles. The pattern was similar across the caste groups. The gap was more among other religions (9% female as 5% male) than Hindus (6% and 4% respectively) in 2005–06.

### Adjusted gender disparity ratio

The adjusted gender disparity ratio was computed using the predicted percentage obtained from the multivariate analysis. The result shown in Table 5 indicates that adjusted gender gap in full immunization was higher in the Central, West, and South regions during 2005–06. More importantly, the gap increased in the West (from 100 to 109) and South (103 to 109) regions during 1992–2006.

**Table 5:** Adjusted† gender disparity ratio in coverage of full and no immunization across the selected characteristics in India, 1992–2006.

	Full immunization			No Immunization		
	1992–93	1998–99	2005–06	1992–93	1998–99	2005–06
Region						
North	124	113	107	136	137	190
Central	123	136	115	117	119	153
East	110	103	103	118	122	92
Northeast	95	128	99	112	107	105
West	100	98	109	101	92	195
South	103	99	109	117	86	103
Place of residence						
Urban	101	101	108	90	107	125
Rural	112	107	107	121	117	130
Household wealth quintile						
Poorest	105	114	107	116	122	110
Poorer	112	100	96	117	107	164
Middle	113	103	105	115	106	105
Richer	106	107	111	115	150	122
Richest	106	103	102	118	115	154
Caste						
SCs/STs	108	109	110	114	115	134
Other	106	103	107	112	115	125
Religion						
Hindu	107	105	107	117	124	136
Muslim	103	105	115	98	91	104
Others	99	101	109	108	109	185

† Based on predicted percentage obtained from multinomial logit regression adjusted for birth order of index child, mother's age at birth of the child, mothers' exposure to media, current working status of mother, father's education, use of antenatal care, and possession of health card.

The adjusted gender gap in full immunization was similar across the rural urban residence during 2005–06. However, the gap is increased in urban areas (101 in 1992–93 to 108 in 2005–06) while decreased in rural areas (112 in 1992–93 to 107 in 2005–06). Across the household wealth quintiles, the adjusted gender gap was highest among the richer quintile (111), followed by the poorest (107) and middle (105) wealth quintiles in 2005–06. It is evident that the disparity has increased among richer wealth quintile while decreased among the remaining quintiles over the study period.

Gender disparity was slightly higher among SCs/STs as compared to other caste groups. However, the gap has remained constant among both the caste groups over the period. Adjusted gender gap in full immunization was highest among Muslims (115), followed by other religions (109) and Hindus (107) in 2005–06. The gap had increased among the Muslims and other religions while it remained the same among Hindus over the study period.

The adjusted gender gap in no immunization coverage was higher in the North, Central, and West regions and the gap increased over the study period. The gap was higher in the urban (125) than the rural area (130) in 2005–06. Moreover, the gap has increased in both urban and rural areas over the study period. The gender gap was highest among poor, rich and richest wealth quintiles. Similarly, the gender gap in no immunization was highest among SCs/STs than other caste groups and it can be seen that the gap has increased across caste groups. Among SCs/STs, adjusted GDR increased from 114 in 1992–93 to 134 in 2005–06 while, among others caste groups; the GDR has increased from 112 to 125 during the same period. The adjusted gender gap in no immunization was highest among other religions (185), followed by Hindus (136), and Muslims (104) in 2005–06. Moreover, the gap has increased across all religious groups over the study period.

## Discussion

Using the data of multi-waves of the NFHS conducted during 1992–2006, our finding shows that the progress in full immunization coverage in the country has been slow and at considerable distance from the goal set by the policy makers. Our finding that girls have lower immunization coverage than boys reinforces the findings of previous studies and surveys, which revealed gender disparities — disfavouring the girl child— in childhood immunization [11], [13], [22]. Trend analysis shows that, at national level, the average gender disparity in full immunization has remained constant. However, when disaggregated, the gender disparity in childhood immunization has increased across the selected socioeconomic contours over the study period.

Gender disparity in immunization coverage was found to be highest in the northern and central Indian states in 1992–93. This could be possibly due to strong preference for sons in these regions [49], [65], [66]. However, we found that the disparity in full immunization had narrowed in the North region whereas it had, astonishingly, increased in the West and South regions of the country over the period. In the western region, the gender disparity ratio in full immunization showed constant increase indicating, thereby, that the increase in vaccination levels was in favour of the male child.

Findings of the study further show that the disparity in no immunization also favoured male children in West and South regions even though the coverage of no immunization remains low in these regions than in the rest of the country. The growing gender disparity in western and southern states can be attributed to increasing preference for sons in these states. Societal attitudes are reflected in declining child sex ratio as in the western state of Maharashtra (from 946 in 1991 to 913 in 2001 and further to 883 in 2011). In the southern state of Tamil Nadu, the sex ratio had declined sharply in 14 out of 32 districts [67]. The results obtained from multivariate analysis also confirm that, after accounting for socioeconomic factors, adjusted GDR in childhood immunization declined in northern and eastern regions whereas it increased in southern and western states.

Gender disparity in full immunization coverage was slightly more in rural areas as compared to urban areas. This could be due to the generally conservative — and traditional — attitude to gender roles in rural as compared to the urban societies. This finding is similar to that of previous study [11]. However, our results appoint to a new trend of increasing gender disparities in both full and no immunization in urban areas. This requires further investigation.

Unlike the widely observed association between poverty and unequal gender relations, our findings do not portray a clear relationship. Gender disparity in full immunization was the worst among richer wealth quintile followed by poorest and middle wealth quintiles. Contrary to that the general perception, girls of the poorer wealth quintile are more likely to be immunized after adjusting for other factors. The disparity either declined or remained constant among the other wealth quintiles, but it increased among the richer quintile during 1992–2006. Previous studies indicated that the gender gap is often more among the lowest wealth quintile [68]. However, these findings show that the gender gap in immunization coverage is a complex issue and pervades all sections of Indian society.

Gender disparity in full immunization was highest among Muslims followed by Hindus and people belonging to other religions. However, the disparity has grown among the Muslims over time. Evidence outside India indicates greater gender based discrimination among Muslim than non-Muslim religion [69]. The greater gender disparity among Muslims can be attributed to an aggregated low level of immunization, which might have act as differing services coverage among Muslim boys and girls. Among Muslims, low education and relatively disadvantaged economic status (due to the patriarchal social setup and ideology) may produce circumstances leading to son being seen as the most dependable socioeconomic insurance [70]. Such a consideration may have resulted in gender discrimination in healthcare utilization among Muslims children.

The extent of gender discrimination in full immunization was slightly higher among children of scheduled castes and tribes than other caste groups. But the gender disparity was much higher for no immunization between these caste groups. It is possible that this gender disparity could be a reflection of the aggregated low level of vaccination coverage among the scheduled caste and schedules tribes [18], [56], [71].

Studies in the 1980's and early 90's showed higher son preferences among higher caste groups as land and property rights were transferred only to male heirs. On the other hand, many tribes that lived in matrilineal social structure showed lower preferences for sons and, consequently, lesser gender inequality [72], [73]. Changing land and property laws have contributed to the decline in gender disparity among higher caste, but appear to have resulted in increasing disparity among SCs/STs recently. Scheduled castes and scheduled tribes are mostly underserved ethnic groups in India. They are mostly landless and without tangible assets. Hence, a male child is preferred as he can be expected to earn a livelihood and contribute to the family income in rural areas.

## Conclusions

Our findings indicate that at aggregate level, the gender disparity in full immunization has stagnated over the 14 years period. However, region-wise and across selected socioeconomic contexts, the gap has widened in many areas. The gender disparity in immunization coverage was greater in northern Indian states. While the disparity has stagnated in the northern states; it has increased in western and southern Indian states. Interestingly, and as a matter of concern, gender disparity has increased in urban areas over the study period. Gender disparity immunization among the middle and upper middle classes has increased over the study period. Findings of this study call for a proper mobilization programme which is required to prevent drop-out from immunization, particularly by families of the girl child. The government and local administration must mobilize community and religious leaders to boost immunization rates and ensure equity in demand for immunization and access by children of both the sexes. As a matter of policy, gender issues must be integrated into India's child immunization programme, particularly in the urban areas, developed states and among Muslims.

### Limitations of the study

Though the study examined the dynamics of gender disparity in childhood immunization across geographical and selected socioeconomic contours in India; however, the findings of the study need to be interpreted with following shortcomings: First, the study was conducted in three rounds in a period of 14 years viz. 1992–2006 which might be a short duration in undermining potential change in gender disparity in immunization coverage. Second, the information on child immunization was collected with the help of health card or mother's reporting where card were not available. The reporting of mother's is prone to recall bias. But, the data used in this study are considered as best and reliable source of information for healthcare in the developing countries.

## Supporting Information

Appendix S1 **Gender gap in full and no immunization among children aged 12–23 months across selected characteristics in India, 1992–2006.**
(DOCX)
