# Supplementary material for: Socioeconomic Dynamics of Gender Disparity in Childhood Immunization in India, 1992–2006
Source: PLoS One. 2014 Aug 15;9(8):e104598. doi: 10.1371/journal.pone.0104598 (PMC4134226; doi:10.1371/journal.pone.0104598)
Supplement: Appendix S1 — Gender gap in full and no immunization among children aged 12–23 months across selected characteristics in India, 1992–2006. (DOCX) [file pone.0104598.s001.docx]

**Appendix S**1 Gender gap in full and no immunization among children aged 12-23 months across selected characteristics in India, 1992-2006.

|  | Full immunization | | | | | | No immunization | | | | | |
| --- | --- | --- | --- | --- | --- | --- | --- | --- | --- | --- | --- | --- |
|  | 1992-93 | | 1998-99 | | 2005-06 | | 1992-93 | | 1998-99 | | 2005-06 | |
|  | Male | Female | Male | Female | Male | Female | Male | Female | Male | Female | Male | Female |
| Region |  |  |  |  |  |  |  |  |  |  |  |  |
| North | 46.8 | 37.7 | 41.4 | 37.0 | 47.5 | 44.4 | 25.1 | 33.0 | 13.1 | 17.3 | 4.2 | 9.3 |
| Central | 24.7 | 19.0 | 22.3 | 16.8 | 30.5 | 26.5 | 38.1 | 44.6 | 23.7 | 27.1 | 3.5 | 4.3 |
| East | 22.5 | 21.8 | 27.1 | 25.7 | 45.8 | 43.5 | 36.7 | 42.7 | 13.2 | 16.2 | 6.4 | 7.7 |
| Northeast | 18.2 | 20.8 | 24.0 | 15.9 | 33.9 | 34.3 | 42.5 | 46.2 | 30.2 | 32.6 | 13.6 | 17.2 |
| West | 57.9 | 60.8 | 66.0 | 64.4 | 56.8 | 50.7 | 11.6 | 11.0 | 3.6 | 3.2 | 2.5 | 4.5 |
| South | 54.8 | 52.8 | 65.0 | 65.8 | 62.6 | 56.8 | 11.0 | 13.2 | 4.0 | 3.5 | 3.7 | 3.8 |
| Place of residence |  |  |  |  |  |  |  |  |  |  |  |  |
| Urban | 49.9 | 51.6 | 54.8 | 54.8 | 59.2 | 55.7 | 17.6 | 15.2 | 6.2 | 6.5 | 2.9 | 4.2 |
| Rural | 32.7 | 29.1 | 36.3 | 33.4 | 40.3 | 36.6 | 30.9 | 37.2 | 15.7 | 18.0 | 5.1 | 6.9 |
| Household wealth quintile |  |  |  |  |  |  |  |  |  |  |  |  |
| Poorest | 18.5 | 16.7 | 19.5 | 17.7 | 25.8 | 22.8 | 43.5 | 50.7 | 24.5 | 28.6 | 8.8 | 10.0 |
| Poorer | 25.8 | 22.5 | 26.6 | 26.3 | 32.7 | 33.8 | 35.0 | 42.3 | 19.6 | 20.4 | 4.6 | 8.1 |
| Middle | 33.2 | 28.8 | 39.8 | 38.1 | 49.1 | 44.5 | 30.4 | 35.3 | 14.7 | 16.0 | 4.4 | 5.2 |
| Richer | 42.8 | 41.6 | 52.3 | 47.7 | 58.4 | 51.4 | 21.6 | 22.2 | 6.3 | 8.4 | 2.3 | 3.6 |
| Richest | 61.2 | 60.2 | 62.6 | 60.2 | 70.4 | 71.8 | 10.1 | 11.6 | 3.2 | 4.1 | 1.0 | 1.0 |
| Caste |  |  |  |  |  |  |  |  |  |  |  |  |
| SCs/STs | 26.5 | 25.1 | 35.6 | 31.8 | 39.2 | 34.8 | 36.5 | 42.2 | 17.0 | 19.3 | 6.3 | 8.9 |
| Others | 39.6 | 36.8 | 43.9 | 42.8 | 54.9 | 52.5 | 25.4 | 29.3 | 11.7 | 13.3 | 3.7 | 4.9 |
| Religion |  |  |  |  |  |  |  |  |  |  |  |  |
| Hindu | 37.3 | 34.7 | 40.9 | 39.0 | 45.8 | 42.8 | 26.2 | 31.1 | 12.2 | 14.7 | 3.7 | 5.6 |
| Muslim | 27.1 | 25.3 | 31.1 | 28.6 | 38.7 | 33.6 | 40.3 | 42.1 | 22.0 | 20.1 | 8.2 | 7.8 |
| Others | 55.4 | 50.2 | 60.7 | 57.8 | 60.7 | 50.7 | 15.6 | 21.8 | 8.9 | 10.7 | 4.8 | 10.0 |
